# lncRNA-Triggered Macrophage Inflammaging Deteriorates Age-Related Diseases

**DOI:** 10.1155/2019/4260309

**Published:** 2019-12-21

**Authors:** Lulingxiao Nie, Peng Zhang, Qian Wang, Xinyi Zhou, Qi Wang

**Affiliations:** ^1^State Key Laboratory of Oral Diseases, National Clinical Research Center for Oral Diseases, West China Hospital of Stomatology, Sichuan University, China; ^2^Department of Prosthodontics, West China Hospital of Stomatology, Sichuan University, China

## Abstract

Aging and age-related diseases (ARDs) share basic mechanisms largely involving inflammation. A chronic, low-grade, subclinical inflammation called inflammaging occurs during aging. Autophagy defects, oxidative stresses, senescence-associated secretory phenotypes (SASPs), and DNA damage generally contribute to inflammaging and are largely regulated by numerous lncRNA through two-level vicious cycles disrupting cellular homeostasis: (1) inflammaging and the cellular senescence cascade and (2) autophagy defects, oxidative stress, and the SASP cascade. SASPs and inflammasomes simultaneously cause inflammaging. This review discusses the involvement of macrophage inflammaging in various ARDs and its regulation via lncRNA. Among macrophages, this phenomenon potentially impairs its immunosurveillance and phagocytosis mechanisms, leading to decreased recognition and clearance of malignant and senescent cells. Moreover, SASPs extracellularly manifest to induce paracrine senescence. Macrophage senescence escalates to organ level malfunction, and the organism is more prone to ARDs. By targeting genes and proteins or functioning as competing endogenous RNA (ceRNA), lncRNA regulates different phenomena including inflammaging and ARDs. The detailed mechanism warrants further elucidation to obtain pathological evidence of ARDs and potential treatment approaches.

## 1. Introduction

Rapid scientific and technological advancements have markedly increased the lifespan of humans, along with an inevitable increase in the prevalence of age-related diseases (ARDs) including cancer and diabetes mellitus, wherein age constitutes the primary risk factor and the prevalence of which increases with age [1]. Although the origin of ARDs has long been investigated, the underlying mechanisms remain unclear. Aging occurs throughout an individual's lifespan [2–4]. Numerous theories have been postulated, among which inflammaging is potentially a prominent contributor to ARDs and an appealing therapeutic target [5].

Inflammaging is defined as a chronic, systematic, low-grade, subclinical proinflammatory environment that accelerates cellular aging [6] and occurs in various ARDs including neurodegeneration-related diseases, metabolic diseases, and atherosclerosis [6, 7]. Cellular senescence and SASPs have been suggested as the two major contributors to inflammaging [8]. Senescent cells produce interleukins, chemokines, growth factors, and proteases, which together constitute the SASP [8, 9]. Macrophages are prototypical immune cells expressing SASPs [10]. SASPs are critical for cell vitality and changes in macrophages. Macrophage's polarization or function are associated with numerous disorders including ARDs [11–13].

lncRNAs regulate different biological phenomena. Numerous putative lncRNAs, which indeed encode micropeptides, have been reported [14], prompting studies on the complexity and importance of these previously disregarded molecules. Advancements in lncRNA-induced ARDs have been reported; however, the mechanism underlying lncRNA regulation of inflammaging remains unknown. This review discusses one potential mechanism wherein lncRNA triggers cellular senescence and the SASP to exacerbate ARDs, thus potentially providing pathological evidence of ARDs and methods for their treatment.

## 2. Macrophage Senescence and Inflammaging: An Intimate Relationship

Cellular senescence is a particularly stable state of permanent cell cycle arrest. Macrophages, although terminally differentiated cells, do not undergo this type of replicative senescence and may hence undergo stress-induced senescence. This concept was first introduced in vitro, and it is widely accepted that stress (including reactive oxygen species [ROS] and autophagy defects) plays an important role in senescence in vivo [15]. Senescence occurs throughout an individual's lifespan and plays diverse critical roles. Senescent cells undergo a characteristic alteration wherein morphological changes, functional impairment, and the expression of senescence-associated *β*-galactosidase (SA-*β*-GAL) and p16^Ink4a^ could be detected [16–18]. For immunocytes including macrophages, aging impairs their capabilities and induces immunodeficiency to some extent [19, 20], leading to reduced immunosurveillance and phagocytosis and resulting in immune evasion among malignant tumors. However, considering the decreased capability of recognizing and clearing senescent cells, the space that normal cells should have occupied is in turn occupied by senescent cells, characterized by a reduction in overall physiological function. Consequently, the organism would be more prone to ARDs.

**Figure 1:**
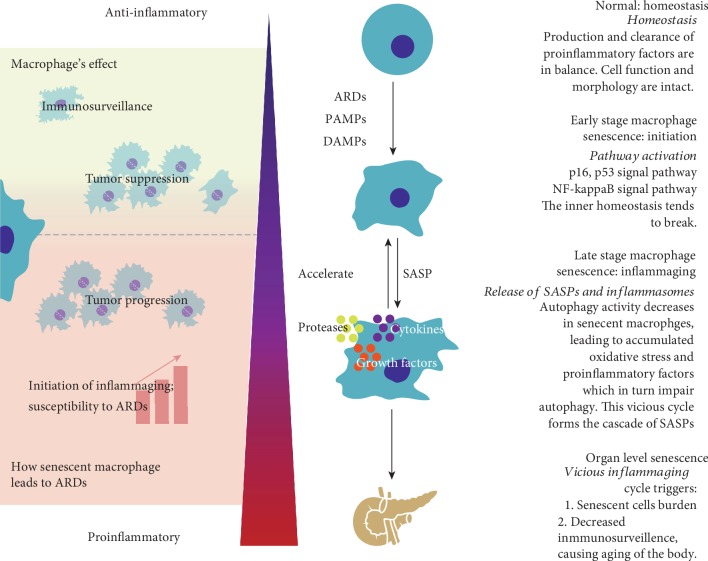
Schematic of the process of cellular senescence and its dual effects.

In healthy conditions, macrophages maintain homeostasis; however, in pathological states, different stresses including DNA damage, telomere shortening, oncogene activation [21, 22], impairment of some key proteins [23], and infections activate the p53, AIM2, and NF-*κ*B signal pathways [24, 25], initiating macrophage senescence. Telomeric DNA is prone to various types of damage including oxidative stress, which can induce telomere shortening [26]. Hence, even terminally differentiated macrophages undergo telomere dysfunction, triggering DNA damage response (DDR) pathways and finally causing cellular senescence [27]. When these damage-associated molecule patterns (DAMPs) and pathogen-associated molecular patterns (PAMPs) are highly intensive or temporally irreversible, the balance between the production and clearance of proinflammatory factors is disrupted. At later stages of macrophage senescence, the net effect is SASP expression (Figure 1). These factors not only aggravate macrophage senescence but are also extracellularly released, thus impairing the functions of surrounding cells. This process is called “paracrine senescence” [28] and causes a wider range of inflammaging. With steady accumulation of senescent cells, senescence eventually occurs at the cellular level and then at the organ level, causing organ malfunction, consequently resulting in corresponding aging phenotypes.

Macrophage senescence is inextricably associated with inflammaging, bridging SASPs and inflammasomes. p16^Ink4a^ has been implicated in macrophage activation or polarization [29], and SASPs induce senescence-like phenotypes in macrophages [30]. Senescence-related changes in macrophages are speculated to generally represent their proinflammatory activation [31], i.e., proinflammatory effects of macrophages are accompanied by inflammaging to some extent. SASPs induce a proinflammatory and aging-promoting environment. This constant detrimental stimulation potentially threatens various cellular components, including organelles and DNA [7]. By increasing SASP expression, these impaired components aggravate inflammaging and consequently impair inflammatory homeostasis, thus accelerating senescence and the susceptibility to ARDs [32]. SASPs and inflammasomes simultaneously cause inflammaging: they trigger inflammaging, thus accelerating aging, and inflammaging is manifested through inflammasome induction and SASP expression. Moreover, autophagy defects, oxidative stress, and DNA damage result in the assembly of inflammasomes and expression of SASPs [31–33].

## 3. lncRNA Triggers Macrophage Senescence in ARDs

Emerging data suggest that lncRNA plays a key role in regulating inflammatory responses. Alterations in various lncRNA expression levels are associated with a proinflammatory phenotype in various ARDs [34–36]. This leads to modification of cellular senescence through several diverse approaches, whether by mediating gene expression or protein function or functioning as competing endogenous RNA (ceRNA). Changes in lncRNAs in ARDs and the corresponding consequences have been widely studied, especially in cancer. However, the association between lncRNA and cellular senescence in ARDs remains an interesting and complex issue. Here, we consider macrophage senescence to investigate the mechanism underlying lncRNA-mediated induction and exacerbation of ARDs.

### 3.1. Diabetes Mellitus

Diabetes mellitus is among the most serious recent public health challenges. Changes in macrophage expression profiles exert local and systemic inflammatory stress. lncRNA E330013P06 regulates proximal genes involved in macrophage functions to increase IL-6, TNF-*α*, and NOS2 levels while downregulating anti-inflammatory cytokines [37]. Besides gene regulation, Lethe and Dnm3os reportedly bind to the p65 subunit and induce epigenetic modifications, thus disrupting nuclear translocation of NF-*κ*B and enhanced inflammatory responses and oxidative stress [38, 39]. Moreover, changes in certain lncRNA expression levels potentially lead to higher, more lethal inflammation [40]. In diabetic complications, MALAT1 potentially triggers pyroptosis in macrophages, thus exacerbating the severe consequences of atherosclerosis [40]. These molecular alterations suggest the disruption of macrophage homeostasis with the direct outcome that pancreatic *β* cells and focal lesions may undergo harsher damage.

### 3.2. Cancer

As shown in Table 1, macrophage-associated cancers are mostly derived from epithelia. A recent theory states that tumor growth depends not only on tumor cells themselves but also on the peripheral cellular and noncellular components [41]. In cancers, macrophages are recruited to the lesion [42]. Such tumor infiltration and the immunosurveillance of macrophages render them an important cell type in cancer. lncRNAs regulate cancer primarily through direct alteration of gene expression. LNMAT1 recruits hnRNPL to the *CCL2* promoter, thus altering its expression and mediating epigenetic alterations and activating and recruiting macrophages to the site of bladder cancer, promoting tumor invasion and lymphatic metastasis [43]. Furthermore, tumor cell-derived lncRNAs are released via exosomes and are internalized by the surrounding macrophages. Changes to downstream pathways disrupt their function, especially phagocytosis, which is critical for tumor clearance [44]. As ceRNA, NIFK-AS1 and CCAT1 decoyed microRNA to suppress macrophage M2 polarization and malignant behaviors [45, 46]. *CCAT1* expression levels differ between M1 and M2 macrophages. Furthermore, lncRNA Cox2 potentially alters M1/M2 polarization, thus preventing immune evasion and metastasis [47]. These reports indicate that macrophage M2 polarization facilitates malignant behaviors and some lncRNAs are essential in maintaining the cellular phenotype.

### 3.3. Atherosclerosis and Related Heart Disease

Atherosclerosis is a chronic inflammatory disease. Macrophages have been recently reported to display marked inflammatory plasticity, particularly polarization. They perpetuate chronic inflammation and growth of atherosclerotic plaques, thus being central to the initiation, growth, and ultimately the rupture of arterial plaques [48]. Studies on atherosclerosis and macrophage have reported that lncRNAs majorly function as ceRNA in causing atherosclerosis. By sequestering microRNAs, MITA, GAS5, HOTAIR, and UCA1 promote M1 polarization, inducing proinflammatory cytokine, matrix metalloproteinase, and ROS levels [49–52]. Furthermore, MeXis and CDKN2B-AS1 interact with DDX17 and DNMT1 to modulate downstream gene expression, thus altering macrophage function and polarization [53, 54]. Atherosclerosis contributes to various lesions, especially cardiovascular disease. Current evidence suggests that the effect of lncRNAs on macrophages in coronary artery disease is the same as that on atherosclerosis [55], highlighting the consistency of its function and prompting its potential as a therapeutic target.

### 3.4. Other ARDs

Besides the aforementioned diseases, lncRNA regulation of macrophage senescence is also reflected in other ARDs, primarily osteoarthropathies. lncRNAs generally function in the same manner in these diseases as in diabetes, cancer, and atherosclerosis. They alter the expression or function of key proteins by targeting genes or proteins or functioning as ceRNAs to disrupt homeostasis and engage in cellular senescence. Although these are indeed phenotypes of aging cells, they are indirect. A study on rheumatoid arthritis reported that NNT regulates downstream gene PBOV-1 via HnRNP-U binding and ultimately alters cellular senescence, i.e., cell cycle arrest [56]. This is a direct testimony to the potential of lncRNA to regulate cell lifespans.

Macrophage polarization is a fundamental phenomenon. Through the mutual transformation of macrophages M1 and M2, macrophages exert opposite functions and participate in different physiological phenomena. M1 polarization is proinflammatory and is associated with inflammaging, while M2 polarization is anti-inflammatory [57]. Increasing evidence indicates that M1 macrophages are at a higher risk of aging-related stress and display senescence phenotypes. lncRNA initiates senescence and ARD pathogenesis by altering macrophage polarization.

## 4. Three Targets of lncRNA to Regulate Macrophage Senescence

lncRNA alters cellular senescence by mediating gene expression or protein function or functioning as ceRNA. Genes regulated by lncRNA during aging are primarily those involved in the p53 pathway [66]. Furthermore, the expression of cellular senescence-related proteins including p21, p27, and p16^Ink4a^ is influenced [67, 68]. lncRNA generally functions in *cis-* or *trans-*action where the former regulates various proximal genes, while the latter regulates distal genes. However, the detailed mechanism underlying lncRNA-gene interactions remains unclear; however, studies have reported certain advancements. lncRNA H19 promotes antiaging effects through miR-675. The latter downregulates p53 and p21 by targeting the 3′UTR of USP10. However, it remains to be elucidated whether there is any mediator in this interplay, along with the mechanism underlying H19 and miR-675 interactions [66]. Furthermore, lncRNA GUARDIN is indispensable for genomic stability by preventing chromosome end-to-end fusion through maintenance of the expression of telomeric repeat-binding factor 2 via sequestering of miR-23a. Moreover, GURADIN downregulation potentially triggers apoptosis and senescence [69]. Certain lncRNAs are involved in genomic stability. R loop formation and cellular senescence have reportedly occurred after alteration of the expression of certain lncRNAs [70]. Moreover, lncRNA CAIF potentially displays transcription factor-like functions trough blocking of p53-mediated myocardin transcription [71].

Along with genes, lncRNA mediates cellular senescence by functioning as ceRNA or by directly targeting proteins. CeRNA competes with microRNAs and regulates their effects on other genes, RNAs, and proteins. lncRNA regulates cellular senescence primarily in this manner. By sequestering various microRNAs, lncRNA prevents them from binding to targets, thus impairing their function [72]. Except for the regulation of gene expression, sponging potentially results in a direct functional change in proteins by attenuating key protein degradation triggered by microRNAs or relieving functional restraints of microRNAs on certain proteins [73, 74].

In a more direct and albeit less frequent manner, proteins become the primary target of lncRNA binding, thus altering their conformation and epigenetic modifications and initiate alterations in their downstream pathways. lncRNA potentially binds to transcription factors or RNA-binding proteins and forms polycomb repressive complexes to regulate downstream genes [75, 76].

## 5. Mechanisms of lncRNA-Induced Macrophage Inflammaging in ARDs

Through three targets, lncRNA manipulates complex biological behaviors. As shown in Table 1, secretome changes induced by lncRNA include SASP expression, i.e., upregulation of matrix metalloproteinase, IL-1*β*, IL-6, and TNF-*α*, and downregulation of IL-4 and IL-10, which contributes to inflammaging and is one of the hallmarks of macrophage senescence [77]. Another notable phenotype is oxidative stress in macrophages. ROS induction is not a rare event in these ARDs. The oxidative stress signal is in a network comprising mitochondria, the endoplasmic reticulum, and numerous mutually regulated signaling pathways. Oxidative stress and autophagy defects are probably the most prominent mutually regulated phenomena. Autophagy inhibition triggered by the induction of oxidative stress [78] causes the accumulation of damaged mitochondria and aggregative proteins and rodex homeostasis disequilibrium, leading to impaired protein-folding capacity, unstable lysosomes, and ROS [32, 79]. This cyclic pathway results in the aging-inducing inflammaging and promotes the expression of SASPs. On combining existing evidence with this theory, lncRNA triggers either ROS, SASPs, or both, thus presenting a self-amplification system targeting genes, microRNA, or proteins to induce inflammaging, which accelerates cellular senescence and eventually leads to the exacerbation of ARDs. This deteriorative cycle occurs not only in disease models but also in the simple aging process [32], highlighting the viewpoint that inflammaging is a ubiquitous phenomenon that boosts senescence in aging or the proaging status and is a promising therapeutic target of ARDs.

**Figure 2:**
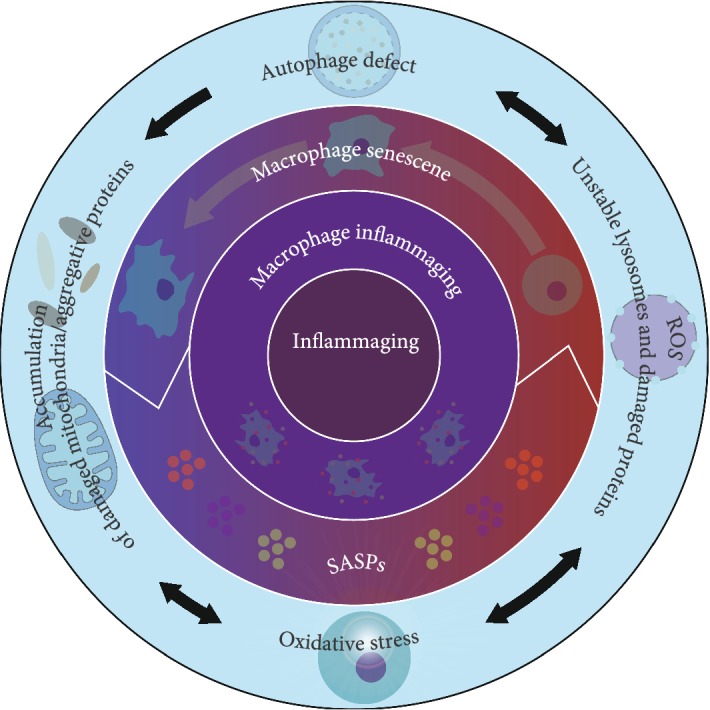
Schematic of the two vicious cycles.

DAMPs and PAMPs induce cellular senescence primarily through the p53 signaling pathway, AIM2 signaling pathway, and the NF-*κ*B signaling pathway [24, 25]. Upon activation, normal cellular homeostasis is prone to disruption in the presence of downstream gene products, wherein the disruption of the balance between production and clearance leads to increased SASP expression and increased inflammasomes. Excessive proinflammatory factors accelerate aging and increase the risk of ARDs. More importantly, SASPs include two vicious cycles, thus forming the cascade (Figure 2): (1) the SASP is an important pathogenic factor and attribute of inflammaging. Therein, IL-1, IL-6, IL-8, MMP-3, and other particular factors can trigger inflammaging, while cells in this proinflammatory condition express SASPs via activation of Rcor2 and the NF-*κ*B signaling pathway [80], resulting in the first deteriorative cycle. (2) Macrophage senescence is inevitably accompanied by a functional decline in organelles. Increasing evidence indicates that autophagy is reduced when cells enter the late stage of senescence [81, 82]. Being at one complex regulatory network comprising numerous other organelles, autophagy defects consequently trigger inflammaging, forming the second deteriorative cycle.

These data undoubtedly depict a mutual association between them and insinuate the significant promoting effects of inflammaging on macrophage senescence. Recent studies on NLRP3 inflammasomes had proved this cascade model [83]. NLRP3 is assembled upon the detection of PAMPs or DAMPs. Thereafter, it activates the corresponding signaling pathway, usually NF-*κ*B, inducing the expression of SASPs and pyroptotic cell death [84]. lncRNA regulates the activation and expression of NLRP3 by sequestering microRNA or inhibiting NF-*κ*B phosphorylation in uric acid nephropathy or in the inflammatory response [85, 86]. Indeed, some studies have reported that inflammaging can cause certain diseases [87], although studies in this field are relatively few. Thus, inflammaging may be a potential target in countering ARDs and delaying aging. Another intriguing aspect is the regulation of the intensity of inflammaging, probably through effects on lncRNAs, such that organisms safely survive the aging process and do not result in disorders [5].

## 6. Conclusion

**Figure 3:**
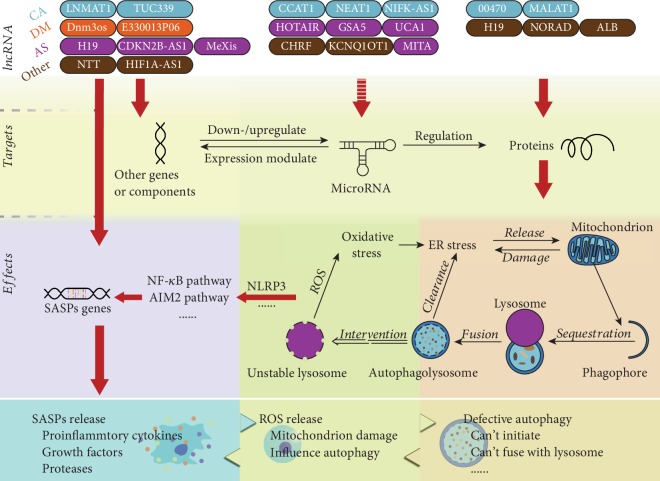
Schematic of the mechanism of how lncRNA triggers inflammaging and cellular senescence. CA: cancer; DM: diabetes mellitus; AS: atherosclerosis.

This review discusses a delicate complement of inflammaging, which emphasizes on the disruption of the cellular onset of systemic inflammatory homeostasis. Increasing evidence indicates that lncRNA regulates inflammation. Alterations in the expression levels of various lncRNAs are associated with a proinflammatory phenotype in various ARDs. By directly binding to proteins or genes or indirectly serving as ceRNA, lncRNA regulates anti-inflammatory and proinflammatory processes (Figure 3). Upon disruption of macrophage homeostasis, SASPs, ROS, and other components would form a self-amplifiable aging-promoting environment to accelerate macrophage senescence and paracrine senescence and worsen ARDs. Defective autophagy, deleterious oxidative stress, and DNA damage SASP expression are the primary pathogenic mechanisms of inflammaging. Their roles are mutual, and it is largely unclear how they trigger inflammaging. Autophagy defects reduce ROS scavenging; ROS accumulation damages mitochondria, resulting in excessive deleterious oxidative stress. Inflammasomes assemble upon the recognition of DAMPs and activate downstream proinflammatory signals, inducing the expression of SASPs, including interleukin and matrix metalloproteinase secretion.

Owing to the marked inflammatory plasticity of macrophage, aberrant activation or functional alterations potentially induce ARDs. The incidence of diabetes mellitus, cancer, and atherosclerosis continues to increase. These ARDs and their complications impose a huge burden on global public health. Although numerous studies have suggested and some have proved the association between macrophage inflammaging and ARDs, the intermediate details warrant further study. Current studies are attempting to harness macrophage inflammaging in ARDs. Future researches require deeper and broader work. Theories put forward in this review link lncRNA to ARDs and summarize in detail the molecular mechanism of this linkage. They also shed light on the relationship of cellular inflammaging and macroscopic senescent phenotypes. These theories would be helpful guidance in finding novel targets for ARDs and other inflammaging-related disorders. Given that new functions of lncRNA are constantly being discovered, they shall always be considered in the pathogenic mechanism of ARDs and more lncRNA shall be found in this process. Furthermore, pharmacotherapeutic intervention of macrophage senescence by senolytic compounds, like metformin, polyphenols, aspirin, and epigallocatechin gallate [88], may be a novel method to prevent or treat ARDs. However, considering the diverse functions of senescent macrophages under different conditions, it remains to be determined whether this intervention is beneficial, probably by utilizing clodronate liposomes or INK-ATTAC [89, 90] to eliminate senescent macrophages.

In conclusion, the effect of lncRNA on macrophage senescence warrants further investigation, with numerous gray areas to be investigated. Targeting lncRNA during inflammaging would be an intriguing and promising approach to understand inflammaging and would help identify methods to treat ARDs.

## Figures and Tables

**Table 1:** Change of lncRNA in different ARDs and their effects and responsible mechanism.

	lncRNA	Disease	Expression of lncRNA in macrophage	Effects	Mechanism	Model	Ref.
Gene	E330013P06	Diabetes mellitus	Increased	Upregulation of IL-6, TNF-*α*, NOS2; downregulation of IL-10	E330013P06 regulate expression of nearby genes involved in macrophage function.	Human Mice	[37]
Protein	Lethe	Diabetes mellitus	Decreased	Production of ROS	Lethe binds to p65 subunit and inhibits the translocation of NF-*κ*B to the nucleus.	Mice	[39]
Protein	Dnm3os	Diabetes mellitus	Increased	Enhancement of inflammation and phagocytosis. Upregulation of IL-6, TNF, NOS2.	Dnm3os promotes proinflammatory function by both *cis-* and *tran-*action, and possibly via changes in epigenetic histone modifications. This is modulated by nucleolin.	Human Mice	[38]
Unknown	MALAT1	Diabetic atherosclerosis	Increased	Induction of pyroptosis	Unknown.	Rats	[40]
ceRNA	NIFK-AS1	Endometrial cancer	Decreased	NIFK-AS1 overexpression suppresses the IL-4-induced M2 polarization of macrophages and malignant behaviors of endometrial cancer.	NIFK-AS1 downregulates miR-146a.	Human	[46]
ceRNA	CCAT1	Prostate cancer	Increased in M1	CCAT1 knockdown promoted M2 macrophage polarization.	CCAT1 targets miR-148a which regulates the expression of PKC*ζ*.	Human Mice (in vitro)	[45]
Gene	TUC339	Hepatocellular carcinoma	Increased	TUC339 reduces IL-1*β* and TNF-*α*, decreases costimulatory molecule expression, and compromises phagocytosis.	Enriched TUC339 in PLC/PRF/5-derived exosomes is internalized by macrophage thus changes downstream pathways.	Human	[44]
Gene	NEAT1	Thyroid carcinoma	Increased	Knockdown of NEAT1 impairs the malignant progression of thyroid papillary carcinoma-1 and inhibits thyroid tumor growth in vivo.	NEAT1 decreases the expression of miR-214.	Human Mice	[58]
Gene	LNMAT1	Bladder cancer	Increased	LNMAT1 promotes invasiveness and lymphatic metastasis, and activates and recruits macrophages into the tumor, inducing lymph angiogenesis.	LNMAT1 recruits hnRNPL to CCL2 promoter leading to increased H3K4 trimethylation and CCL2 expression.	Human Mice	[43]
Unknown	Cox2	Hepatocellular carcinoma	Increased in M1	Change of Cox2 alters M1/M2 polarization, regulating immune evasion and metastasis of HCC.	Unknown.	Mice	[47]
ceRNA	MIAT	Atherosclerosis	Increased	Knockdown of MIAT attenuates atherosclerosis progression, promotes atherosclerotic plaque stability, and improves efferocytosis.	MIAT acts as ceRNA to positively regulate CD47 expression by sponging miR-149-5p.	Human Mice	[52]
ceRNA	GSA5	Atherosclerosis	Increased	Upregulation of IL-6, IL-1*β*, TNF-*α*, MCP-1, MMP-2, MMP-9.	GSA5 directly binds and suppresses miR-221 expression.	Human	[51]
ceRNA	HOTAIR	Atherosclerosis	Increased	HOTAIR greatly increases total cholesterol, triglyceride levels, dil-ox-LDL uptake rate, ROS, IL-6, IL-1*β*, TNF-*α*, and Cox2 levels.	HOTAIR serves as a sponge of miR-330-5p.	Human	[50]
ceRNA	UCA1	Atherosclerosis	Increased	UCA1 increases ROS and cell apoptosis.	UCA1 sponges miR-206.	Human	[49]
Gene	H19	Atherosclerosis	Increased	H19 increases TG, TC LDL-C, IL-1*β*, TNF-*α*.	H19 upregulates the expression of miR-130b.	Human Mice (in vitro)	[59]
Protein	MeXis	Atherosclerosis	Increased	Loss of MeXis impairs macrophage Abca1 expression and accelerates atherosclerosis.	MeXis interacts with and guides promoter binding of the transcriptional coactivator DDX17 to amplify gene *Abca1*.	Human Mice	[54]
Protein	CDKN2B-AS1	Atherosclerosis	Decreased	CDKN2B-AS1 inhibits inflammatory response and promotes cholesterol efflux.	CDKN2B-AS1 binds to DNA methyltransferase 1 to enhance methylation of ADAM10 promoter which inhibits AMAM10.	Human Mice	[53]
Unknown	ENST00000444488.1 and Uc010yfd.1	Coronary artery disease	Increased (ENST00000444488.1); decreased (Uc010yfd.1)	Silencing of ENST00000444488.1 and Uc010yfd.1 decreases or increases proinflammatory cytokines, respectively.	Unknown.	Human	[55]
Unknown	NEAT1	Myocardial infarction	Increased	Knockdown of NEAT1 disturbs monocyte-macrophage differentiation and its function.	Unknown.	Human Mice	[60]
ceRNA	CHRF	Pulmonary fibrosis	Increased	CHRF promotes silica-induced pulmonary fibrosis and upregulates IL-1*β*, TGF-1*β*.	CHRF negatively regulates miR-489.	Human Mice	[61]
ceRNA	KCNQ1OT1	Osteolysis	Decreased in M1	Overexpression of KCNQ1OT1 induces M2 polarization to ameliorate PMMA-induced osteolysis.	KCNQ1OT1 functions as a miR-21a-5p decoy which regulates IL-10 expression.	Mice	[62]
Gene	HIF1A-AS1	Liver failure	Increased	HIF1A-AS1 promotes TNF*-α*-induced apoptosis.	HIF1A-AS1 upregulates the expression of caspase.	Mice (in vitro)	[63]
Protein	NTT	Rheumatoid arthritis	Increased	Rheumatoid arthritis lead to overexpression of PBOV-1 in macrophage, resulting in cell cycle arrest.	NTT regulates downstream gene *PBOV-1* via HnRNP-U binding and is regulated by C/EBP*β*.	Human	[56]
Unknown	H19	Rheumatoid arthritis; osteoarthritis	Increased	Unknown.	Unknown.	Human	[64]
Unknown	HOTAIR	Rheumatoid arthritis	Increased	HOTAIR induces the migration of active macrophage.	Unknown.	Human	[65]
